# Thermal Response Analysis of Phospholipid Bilayers Using Ellipsometric Techniques

**DOI:** 10.3390/bios7030034

**Published:** 2017-08-18

**Authors:** Carmen M. González-Henríquez, Vanessa A. Villegas-Opazo, Dallits H. Sagredo-Oyarce, Mauricio A. Sarabia-Vallejos, Claudio A. Terraza

**Affiliations:** 1 Departamento de Química, Facultad de Ciencias Naturales, Matemáticas y del Medio Ambiente, Universidad Tecnológica Metropolitana, P.O. Box 9845, Correo 21, Santiago 7800003, Chile; vane_alejandra@msn.com (V.A.V.-O.); dhsagredo@uc.cl (D.H.S.-O.); 2 Departamento de Ingeniería Estructural y Geotecnia, Escuela de Ingeniería, Pontificia Universidad Católica de Chile, P.O. Box 306, Correo 22, Santiago 7820436, Chile; masarabi@uc.cl; 3 Facultad de Química, Pontificia Universidad Católica de Chile, P.O. Box 306, Correo 22, Santiago 7820436, Chile; cterraza@uc.cl

**Keywords:** binary phospholipid mixture, heterogeneous membranes, thermal phase transitions, ellipsometry, surface plasmon resonance

## Abstract

Biomimetic planar artificial membranes have been widely studied due to their multiple applications in several research fields. Their humectation and thermal response are crucial for reaching stability; these characteristics are related to the molecular organization inside the bilayer, which is affected by the aliphatic chain length, saturations, and molecule polarity, among others. Bilayer stability becomes a fundamental factor when technological devices are developed—like biosensors—based on those systems. Thermal studies were performed for different types of phosphatidylcholine (PC) molecules: two pure PC bilayers and four binary PC mixtures. These analyses were carried out through the detection of slight changes in their optical and structural parameters via Ellipsometry and Surface Plasmon Resonance (SPR) techniques. Phospholipid bilayers were prepared by Langmuir-Blodgett technique and deposited over a hydrophilic silicon wafer. Their molecular inclination degree, mobility, and stability of the different phases were detected and analyzed through bilayer thickness changes and their optical phase-amplitude response. Results show that certain binary lipid mixtures—with differences in its aliphatic chain length—present a co-existence of two thermal responses due to non-ideal mixing.

## 1. Introduction

The detection of biomacromolecules (like viruses, exosomes, liposomes, and proteins) in solid-liquid interfaces is an important topic that is currently being studied by several research groups [1,2]. The most common way of detecting these macromolecules is via optical biosensors, which serve as a support for qualitative and quantitative analysis; measuring the direct, real-time and label-free detection of various biomolecular and chemical substances [3,4]. The characterization of the dynamic behavior of the macromolecule adsorption process could generate fundamental knowledge that can be applied in a wide range of medical applications like health-care diagnostics and drug delivery/synthesis, among others [5,6]. According to the study performed by Cho et al. [7], the environmental temperature affects the phospholipidic vesicle adsorption deposited on titanium oxide substrates. The vesicle deformation was characterized at two different temperatures (23 °C and 50 °C) by using localized surface plasmon resonance (LSPR).

According to this, the thermal characterization of artificial phospholipidic bilayers is fundamental to fully understand the adsorption process and, therefore, the sensing capacity of bilayer-based biodevices. To investigate this, further, the development of integral and stable biomimetic membranes has been a topic widely analyzed by different research groups during the past years. These studies have been focused mainly on the modeling of native bilayer membranes with the finality of understanding their behavior under certain conditions and, obviously, with the aim of finding possible scientific applications [8,9]. These studies lead to interesting discoveries such as the strong interaction between anionic phospholipids monolayer and different bisphenol molecules detected in the environment, opening the opportunity to develop biosensors based on phospholipid bilayers that could detect this organic synthetic compound which produces several risks for health, due to their polluting effect. 

Other studies have been mainly focused on the artificial membrane formation and its possible technological applications: anti-cancer drugs binding (insertion/accumulation), interaction with antimicrobial peptides and voltage-gated channels [10,11,12]. As it is possible to appreciate, multiple utilities for these structures have been proposed, relevant for several biomedical and biotechnological applications. These studies coincide in the fact that is necessary to find a method which produces stable self-assembled phospholipid bilayers that can resist important changes in environmental conditions and remain in a functional state [13,14]. 

For example, Skjevik et al. demonstrated that a self-assembled phospholipid structure forms a spontaneous stable lamellar bilayer in few microseconds in an aqueous environment with a clear preferential orientation with polar head groups pointing outside [15]. However—experimentally—the bilayer packing is related to full water hydration (Van der Waals interactions) through hydrogen bridges [14,16,17,18,19] and lipid interactions [20]. Recently, Brea et al. showed that a reversible chemoselective reaction can be harnessed to achieve non-enzymatic spontaneous remodeling of the phospholipid membrane [21]. 

The structures of lipid bilayers at an atomic resolution were theoretically and experimentally studied by Ollila and Pabst [22]. Thus, the C-H bond order parameters, spin relaxation rates, and scattering form factor were fully characterized via dynamic simulations (MD), Nuclear Magnetic Resonance (NMR) and small-angle X ray or neutron scattering (SAXS/SANS) experiments. The conclusion of these studies was that the segmental order parameter and spin relaxation rates are related to the sample structure and dynamics of individual molecules, while that scattering form is related to the average structures of the whole bilayer. These results were corroborated theoretically using MD simulations. However, the interaction described in MD models need to be improved in order to resolve the atomistic resolution properties of glycerol backbone and choline regions. Additionally, Pabst et al. study the coexistence of liquid-ordered, L_α_ (ordered) and liquid-disordered, L_α_ (disordered) domains in fully hydrated multilamellar vesicles by using SAXS, allowing them to determinate slight differences in membrane thickness, area per lipid, hydrocarbon chain length and bending fluctuations in mixtures of 1,2-dioleoyl-*sn*-glycero-3-phosphocholine (DOPC), 1,2-distearoyl-*sn*-glycero-3-phosphocholine (DSPC), 1,2-dipalmitoyl-*sn*-glycero-3-phosphocholine (DPPC) and cholesterol. The main conclusion of this study was that the increase of cholesterol concentration produce important changes in structural properties of L_α_ (disordered), while that the influence in L_α_ (ordered) was marginal [3]. 

Most of the mammalian cell membranes are composed of different lipid classes, establishing phosphatidylcholines (PC) as the main structural compound, distinguishing DPPC and 1,2-dimyristoyl-*sn*-glycero-3-phosphocholine (DMPC) as common models for study [23]. PC-type molecules have different characteristics and intrinsic properties such as saturation degree, hydrocarbon chain length, chemical details of the polar head groups, and so on. Modifications of the bilayer structure should be related to the molecular stability of their components as a function of the temperature [24,25] or pressure changes [26] that the environment suffers gradually. Other studies based on PC phospholipids are, for example, the incorporation of melatonin into bilayers of DSPC, a study that was mainly focused on the development of therapeutic advanced systems [27]. Another example is the mixture of this compound with DOPC that could be mainly utilized in mycotic treatment [28]. 

Most of the biological membranes in cell types, including human erythrocytes and a certain strain of Gram-positive bacteria, possess an asymmetric trans-bilayer phospholipid distribution [29,30], being essential for normal membrane function (transduction, membrane fusion, and cell apoptosis). The disruption of this uncommon structure is associated with the physiological or pathological consequences of difficult medical treatment [31]. The morphological and structural effect of lipid translocation (or “flip-flop” as it is affectionately known) has also been studied by the research groups of Conboy [32] and London [33]. The “flip-flop” effect is common in asymmetric bilayers and influences the bilateral distribution of cellular membrane components in eukaryotic cells. There are several chemical and physical directors which govern the kinetic rates and activation thermodynamics of lipid translocation, and some of these could affect the lipid structure, packing degree and inclusion of cholesterol or transmembrane peptides in the lipid bilayer. Accordingly, it becomes important to study the thermodynamic behavior of asymmetric lipid bilayers. Other studies performed by London [34,35] demonstrate that the chemical composition of asymmetric bilayers also influents the formation of ordered lipid rafts or ultra-nanodomains (<5 nm of radius), showing that membranes with a composition close to mammalian cells (brain sphingomyelin (SM), 1-palmitoyl 2-oleoyl phosphatidylcholine (POPC) or DOPC/DPPC bilayers with higher cholesterol concentration) tends to form ultra-nanodomains in preference to larger domains over the widest temperature range.

Interestingly, phospholipid-cholesterol ternary phase diagrams, composed by a heterogeneous mixture of POPC, N-palmitoyl sphingomyelin (PSM) and cholesterol, showing a heterogenic coexistence of different phases—liquid ordered and disordered, among others—over a wide range of composition ratios and temperatures, have recently been reported [36]. Recently, methyl-β-cyclodextrin mediated lipid exchange technique has been reported as a suitable method for preparing small unilamellar vesicles (SUVs) with stable asymmetric lipid composition [37]. 

In the present work, we have extended our previous thermal studies to two kinds of phospholipids (DPPC and DSPC) and their mixture at different ratios with DMPC or DOPC. These mixtures produce heterogeneous membranes and the co-existence of different phospholipid phases. Ellipsometric phase angle variations (Δ and ψ) of a red laser light after sample reflection is associated with small perturbations in the morphology or roughness of the bilayer. Additionally, the incidence angle variation (Θ) utilized for Surface Plasmon Resonance (SPR) method can be deduced and used to determine the possible phase/phase transition temperature of each compound in study.

The artificial phospholipidic bilayers with homogeneous and heterogeneous compositions were supported over hydrophilic silicon wafer—previously treated—and deposited by Langmuir-Blodgett technique. According to Crane et al. [38,39], this deposition method ensures the formation of an asymmetric bilayer, but in this study, no sign of asymmetric bilayer formation can be asseverated by using ellipsometric techniques. 

Molecular movements, inclination degree and intramolecular interactions into the bilayer were monitored and analyzed in respect to the extern temperature applied. The aim of this study is to understand the effect that saturation and aliphatic chains length produce in membrane stability when the system is subjected to repetitive heating cycles. Results suggested the co-existence of phospholipidic phases, which are related to the heterogeneous compositions and to the length difference of the phospholipids utilized. 

Small variations in the layer were detected and analyzed through slight thickness changes and phase-amplitude response using Ellipsometry and Surface Plasmon Resonance (SPR) techniques, respectively. For this last characterization method, the same ellipsometric equipment was used as a base, but the assembly was slightly modified, including a dove prism into the optical path that permits to obtain a higher resolution and sensitivity in SPR signal detection [40].

## 2. Materials and Methods

### 2.1. Materials

1,2-dipalmitoyl-*sn*-glycero-3-phosphocoline semisynthetic (≥99%, DPPC), 1,2-distearoyl-*sn*-glycero-3-phosphocholine (≥99%, DSPC), 1,2-dimyristoyl-*sn*-glycero-3-phosphocholine (≥99%, DMPC) and 1,2-dioleoyl-*sn*-glycero-3-phosphocholine (DOPC) lyophilized powder were acquired from Sigma-Aldrich Company (St. Louise, MO, USA) and used without further purification. Chloroform (99.0–99.4%), hydrogen peroxide (30–32%), sulfuric acid (95–97%, Emparta^®^ACS) and water LiChrosolv^®^ for chromatography were obtained from Merck KGaA (Darmstadt, Germany). 

Immersion oil for microscopy (UN 3082-9, PG III) was purchased from Merck Millipore (Billerica, MA, USA). Uncoated N-BK7 right angle prism (15 mm) was acquired from Edmund Optics (Barrington, NY, USA). Gold targets of 99.99% purity (Ø 57 mm × 0.2 mm) for argon sputter deposition was obtained from Ted Pella products (Redding, CA, USA). 

A p-type <100> orientation silicon wafer was acquired from Siegert Wafer GmbH (Aachen, Germany). Substrate characteristics are: resistivity 10–20 Ω cm, thickness 525 ± 20 µm, diameter 100 ± 0.3 mm, prime grade, CZ growth (Czochralski process), boron doping (P/B type), Single Side Polishing (SSP) with 2 Semi-standard flats, Total Thickness Variation (TTV) <5 µm, bow <30 µm, warp <30 µm, particles < 10 of 0.3 µm. 

### 2.2. Equipment

Artificial membrane deposition over the silicon wafer was achieved using a Langmuir-Blodgett trough model LT-103 (MicroTestMachines) following the Y-type deposition procedure.

A Multi-angle laser ellipsometer model SE400Adv, from SENTECH Instrument GmbH (Berlin, Germany), was used to perform optical measurements with variable incidence angle from 30° to 90° in steps of 0.5°; the equipment possesses an attached motorized goniometer from Hüber Diffraktionstechnik GmbH & Co. (Rimsting, Germany) for control incidence angle variation. A stabilized He-Ne laser (λ = 633 nm) permits a precision of ±0.1 Å in thin films thickness measurement. In addition, this instrument was used in conjunction with a home-made copper sample holder which facilitated heat conduction. The ellipsometer is coupled with a temperature controller model 325, from Lake Shore Cryotronics Inc. (Westerville, OH, USA), used for regulate surface temperature between 25 °C and 70 °C. A Pt_100_ temperature sensor (model PT-102-2S, useful range 1.4 K to 873 K) and a 25 Watt heater (model HTR-25) were used to close the temperature loop, both devices purchased from Lake Shore Cryotronics Inc. (Westerville, OH, USA). 

An Atomic Force Microscope model NaioAFM from Nanosurf GmbH (Langen, Germany) was used for obtaining surface micrographs and force spectroscopy measurements; the last data permit to determine the Young modulus of the artificial phospholipid bilayers by using the Hertz model for spherical tip indentation over a soft surface [41,42]. 

An argon sputter coater (Cressington Scientific Instrument UK, 108 AUTO, Watford, UK) coupled with a high-resolution film thickness monitor (Cressington Scientific Instrument UK, MTM-20, Watford, UK) with a rotary tilting stage (with 90 rpm frequency) was used to deposit 40 ± 0.01 nm gold over one side the BK7 dove prisms. When the red laser excites the atoms in gold layer, the surface resonance effect is generated, a condition necessary for the execution of the SPR technique. 

### 2.3. Substrate Preparation

Substrates—silicon wafer (100)—needs to be pre-treated using a piranha solution in order to obtain a hydrophilic surface through the remotion of the native silicon dioxide film. The mixture is composed by H_2_SO_4_:H_2_O_2_ (7:3 *v/v* ratio); the substrates were immersed into the solution during 30 min. in a thermal bath at 80 °C. Afterwards, the silicon wafers were washed with Milli-Q water and then dried with an ultra-pure nitrogen gas jet [43]. This method generates a SiO_2_ layer of thickness near to ~2.1 nm and, consequently, a low contact angle near to ~10°, a necessary condition for deposit bilayers over the substrate. 

### 2.4. Surfactant Deposition Using Langmuir Blodgett Technique

Fully-saturated lipid monolayers were deposited onto silicon wafers. These depositions were performed using optimal deposition speed; slow enough to allow liquid drainage and fast enough to prevent hole formation [44]. 

The deposition process was initiated with the hydrophilic silicon wafer immersed in a trough full of Phosphate Buffered Saline (PBS, pH 7.4). Bilayer phospholipid deposition procedure was as follows: DPPC (1 mg), DSPC (1 mg), DPPC:DMPC (2:1, 1.5 mg) or DPPC:DOPC (7:1, 1.5 mg) were dissolved in 0.5 mL of chloroform. This solution was then spread drop by drop with a Hamilton syringe (60 μL of solution) over PBS interface (Langmuir-Blodgett trough) at constant room temperature (21 °C) with subsequent solvent evaporation for a few minutes. Secondly, their isotherm was measured using a compression rate of ~1.5 mm/s, enough velocity that allows DPPC monolayer formation in the interface. The compression arm moved at this velocity until reach the appropriate surface pressure for deposition (~45 mN/m) [45]. Then, the substrate was pulled upwards slowly at 0.01 mm/s in order to form a homogeneous monolayer with high coverage over the surface, without the presence of pores; this procedure takes close to 30 min, their homogeneity was monitored via AFM micrographs in a previous study performed by our research group [24]. Subsequently, the substrate was slowly immersed into the interface for a second deposition. At this point, in order to avoid third layer deposition, a barrier was moved to the original position for surfactant remotion from PBS interface with a specialized liquid vacuum equipment; finally, sample emersion occurred. Using this protocol is possible to generate a homogeneous planar phospholipid Y-type bilayer over the silicon substrate.

This deposition method ensures an asymmetrical labeled bilayer with 70–80% of asymmetry once the procedure was completed, according to the research group of Crane [38,39]. These conclusions were obtained by using fluorescence interference contrast microscopy (FLIC). But, in this study, no clear signs of trans-bilayer asymmetry can be observed, due to the optical characterization techniques used.

### 2.5. SPR/Ellipsometry Set-Up

The commercially multi-angle laser ellipsometer model SE400Adv, from SENTECH Instrument GmbH (Berlin, Germany), was used as a base for Ellipsometry and SPR assemblies. For SPR, a layer of gold (40 ± 0.01 nm gold) needs to adhere to one surface of a dove prism. This metal acts a waveguide for microwave or optical frequencies of the incident light [46]. The film thickness should be higher than the cut-off thickness, necessary for plasmonic generation, which is around λ/2 (λ is the excitation wavelength). Additionally, the films need to be surrounded by materials with lower refractive indices in order to enable the wave propagation by total internal reflection in the metallic material [47]. Finally, the naturally generated silicon dioxide layer over the silicon wafer used as a substrate is commonly used as an additional waveguide which displays very good hydrophilicity, thus offering a suitable platform for lipid vesicle fusion and biomembranes analysis. 

**Figure 1:**
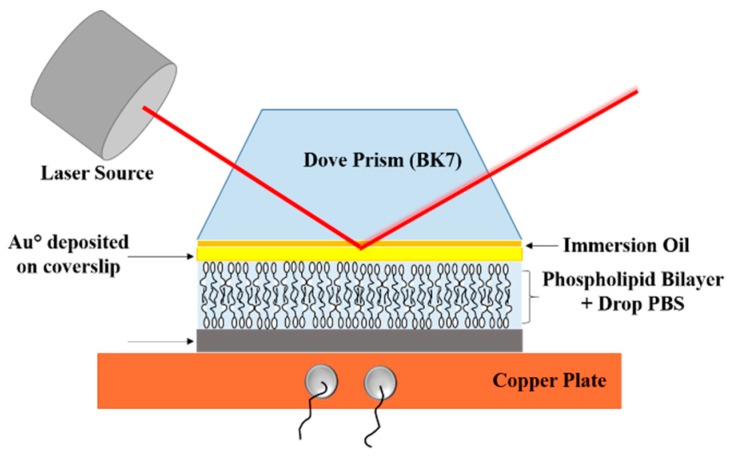
Schematic diagram of Surface Plasmon Resonance (SPR) assembly used for the experiments.

Through the so-called Kretschmann optical configuration is possible to assemble the SPR technique based on an ellipsometric equipment [48]. This configuration includes a laser source, polarizers, compensator, a heating sample stage and a sensitive response detector. Figure 1 shows the incident and reflected rays that interact with the prism; it is also possible to observe the adhered gold layer which is found in direct contact with the artificial bio-membrane. The technique proposed in this study is very similar to that explained by Moirangthem et al. [49] with the difference that our system is able to measure small SPR signal variations in a semi-dry environment without the presence of a liquid chamber. This small variation in the assembly allows us to precisely modulate and control the temperature, permitting to evaluate the thermal behavior of the phospholipid bilayers. Is important to mention that the artificial biomembranes must be previously moisturized with a drop of PBS in order to avoid denaturalization of the components.

### 2.6. Ellipsometric Model and Refractive Indexes

The physical properties (thickness) of the system were measured by ellipsometry through an optical model designed for thin slab layers based on the assumptions first made by Drude, which have been subsequently adjusted over the years [24]. The equipment possesses the PCSA (Polarizer-Compensator-Sample-Analyzer) optical path, in which a monochromatic red laser (He-Ne, λ = 633 nm) first passes through a polarizer, secondly by a compensator, then the laser impacts over the sample and reflects it until reaching the analyzer, finally impacting the detector. The polarizer and the analyzer automatically rotate in order to obtain near null-intensity on the detector. This kind of system permits the detection of minimal changes in the ellipticity (polarization states) of the non-normal light reflected from the sample, allowing to characterize slight variations in slab system thickness. 

The software automatically simulates a parametric curve which permits to estimate the expected polarization angles (Ψ and Δ) depending on the thickness and refractive index of the analyzed layer based on the modified Drude model. These polarization angles have a direct relation with the measured angles in the polarizer and the analyzer. Using these values—and the parameters obtained with the simulated optical model—the thicknesses of the layers can be precisely determined if the refractive index is known.

In our case, the refractive indexes were measured as a solution and then as a layer over the substrate through an Abbe refractometer. The refraction indexes measured in the different configurations are tabulated in Table 1. Using the same procedure, the refraction indexes of the solution at higher temperatures were also measured; the results show that the refraction indexes slightly decrease from 1.489 at ambient temperature (22 °C) to 1.476 at 55 °C, for the case of DPPC. A similar behavior presents the rest of the phospholipidic solutions. 

## 3. Results and Discussion

### 3.1. Thermal Studies Performed by Ellipsometric and SPR Technique

**Figure 2:**
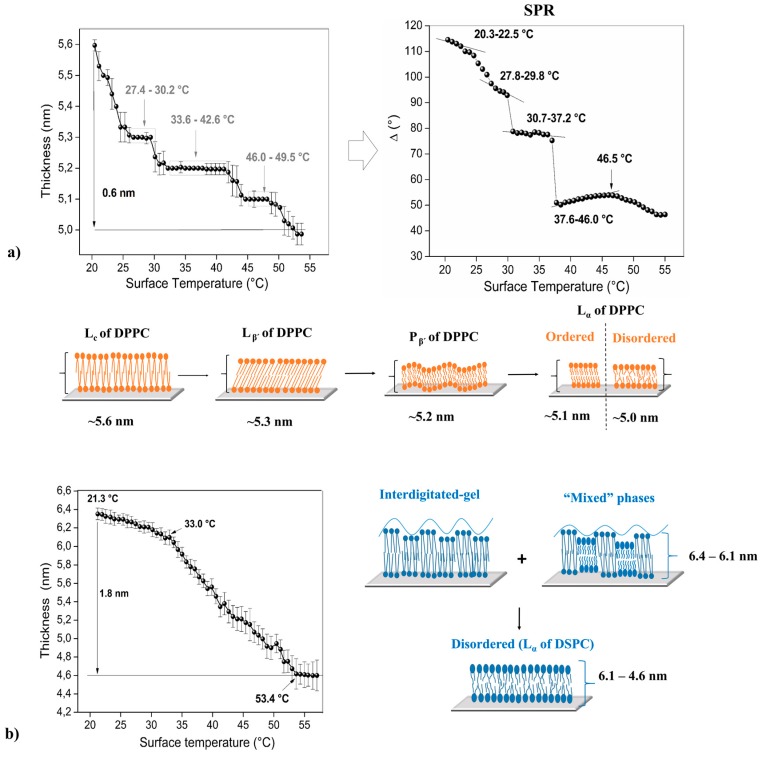
Optical response and some schemes of the bilayers measured under heating cycles for (**a**) 1,2-dipalmitoyl-*sn*-glycero-3-phosphocholine (DPPC) (ellipsometry and SPR) and (**b**) 1,2-distearoyl-*sn*-glycero-3-phosphocholine (DSPC) (ellipsometry).

**Figure 3:**
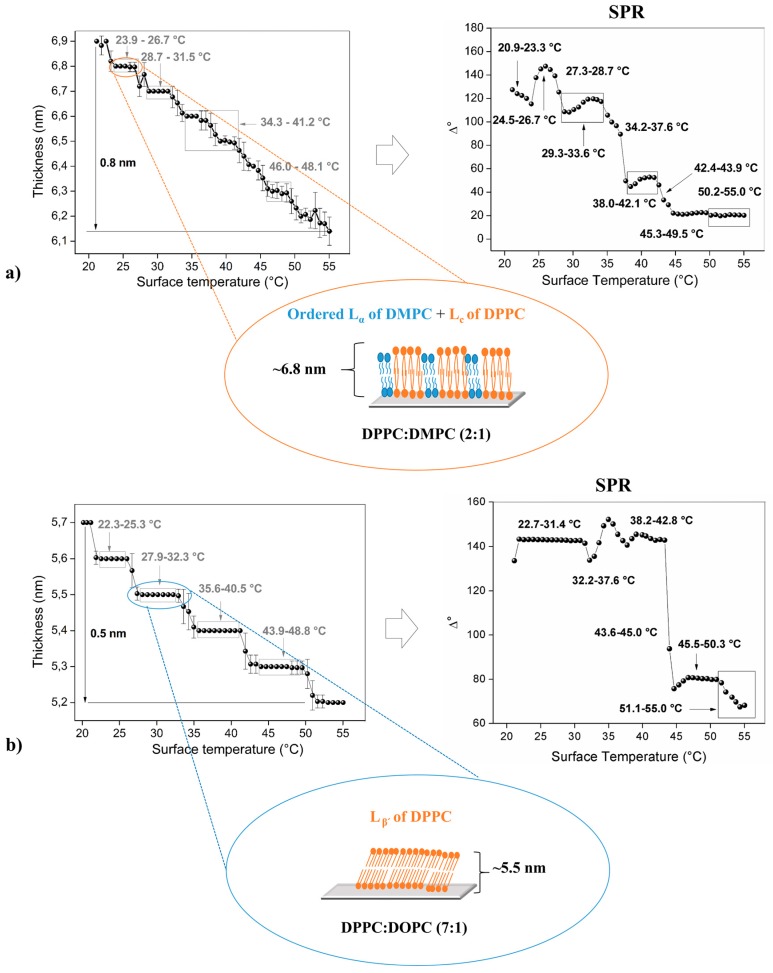
Thermal behavior detected by optical studies (ellipsometry and SPR) with their respective diagrams for (**a**) DPPC: 1,2-dimyristoyl-*sn*-glycero-3-phosphocholine (DMPC) (2:1) and (**b**) DPPC: 1,2-dioleoyl-*sn*-glycero-3-phosphocholine (DOPC) (7:1).

**Figure 4:**
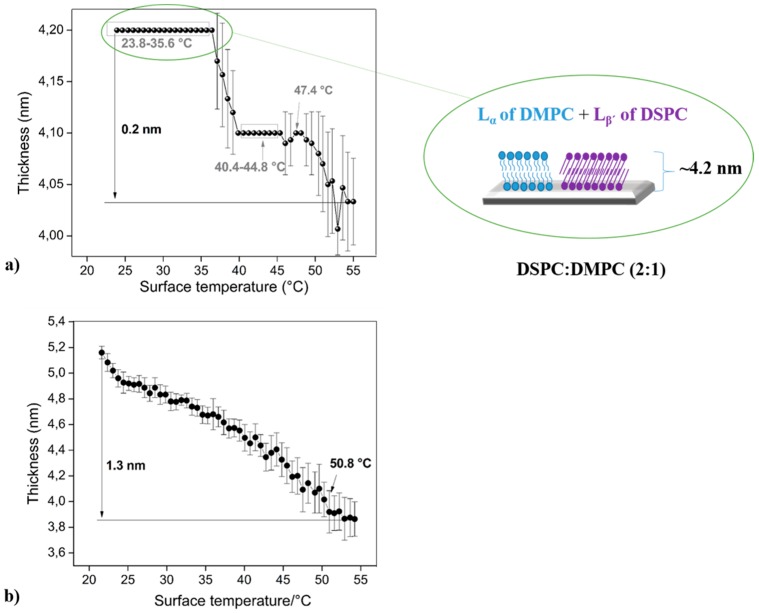
Thermal behavior detected for optical studies (ellipsometry) and a diagram of (**a**) DSPC:DMPC (2:1) and (**b**) DSPC:DOPC (7:1).

Figure 2, Figure 3 and Figure 4 shows the thermal behavior of the analyzed phospholipidic bilayers composed by pure surfactants and its mixtures (heterogeneous membranes). The systems analyzed in this study represent common heterogeneous structures that possess different physical characteristics such as chain length and simple/double bonds presence in the aliphatic chain. Optical ellipsometry parameters (angles Δ and φ) were monitored under controlled heating cycles, allowing to calculate thickness changes respect to the applied temperature. DPPC, DPPC:DMPC and DPPC:DOPC mixtures were studied by SPR technique, permitting the recognition of their molecular movements when they are subjected to stressful conditions, allowing the detection of minimal changes in their refractive index, a characteristic that is intrinsically related to molecular interactions generated into the membrane (Figure 2 and Figure 3). The error bars in the ellipsometry plots are associated with the standard deviation generated after thirty continuum measurements (1 per s) performed under the same experimental conditions. The SPR data was also obtained by thirty continued measurements, so the standard deviation of this data can also be determined, but in this case, the error bars were not placed in the graph because there are extremely small, making their clear appreciation difficult. 

### 3.2. Pure Phospholipid Bilayer—Homogeneous Membrane

Transitions and phase transitions of the artificial phospholipidic bilayers were detected via ellipsometry technique using controlled heating cycles (Figure 2, Figure 3 and Figure 4). Thickness was estimated through the fitting of these parameters into an optical model based on Drude assumptions. 

Parallel, Δ values were obtained using SPR configuration, these results can be observed in Figure 2 and Figure 3. Variation of the angle Δ is more significant than the variations of φ, this effect occurs because the optical phase response is much more sensitive than the response of the amplitude at this range of thickness; less than 20 nm [49]. The optical measurements (SPR and ellipsometry) of the different surfactants was performed in a moisture environment using a drop PBS solution prior to measurement. This point is fundamental in order to maintain the humectation of the sample, necessary to ensure a bilayer structure and avoiding component denaturalization [50]. It is noteworthy that the optical and physical properties of the gold layer deposited over the prism for SPR measurements—such as refraction index and thickness—do not depend on temperature applied for the study (ranged from ~25 °C to 70 °C), taking into consideration the thermal expansion, the electron-phonon scattering of the Au microparticles and the dielectric permittivity of the host matrix [51]. 

Figure 2a shows an initial DPPC thickness of 5.6 nm at 21.4 °C measured with ellipsometric technique. This value is related to preferred molecular orientation after its deposition on the substrate, this parameter decay to 5.0 nm at ~55.0 °C (differential thickness: 0.6 nm). At low temperatures, the phospholipidic chains are packed very tightly and the rotation about their long axis is restricted to steric and Van der Waals interchain interactions. Generally, the phospholipidic bilayers present different phases and phase transitions when the sample is subjected to heating cycles, in this case, the aliphatic chain of DPPC shown certain interdigitation with a subsequent bilayer fluidity at higher temperatures (see scheme in the Figure 2) [24]. 

In the first cycle (Figure 2) is possible to detect three plateaus between 27.4–30.2 °C, 33.642.6 °C and 46.0–49.5 °C, which should be related to L_β’_, P_β’_ and L_α_ (ordered) phases, respectively. In addition, two phase transitions were detected (L_β’_–P_β’_ and P_β’_–L_α_), located at 30.8–32.9 °C and 43.2–45.4 °C, respectively. Finally, the data acquired between 50.1 and 55.0 °C suggest the presence of an unstable bilayer structure, L_α_ (disordered) phase, indicated mainly by the higher statistical error in the measurements. Figure 2a (right) shows some of the results obtained via SPR data (Δ *v/s* T); in this case, the slope change could be associated with the variation of the molecular orientation, packing densities or spatial homogeneity of the system analyzed (phospholipid bilayer). Thus, the same phases and with similar temperature phase transitions were detected in this case: 20.3–22.5 (L_C_); 23.0–27.2 (L_C_–L_β’_); 27.8–29.8 °C (L_β’_); 29.8–30.7 °C (L_β’_–P_β’_); 30.7–37.2 °C (P_β’_); 37.2–37.6 °C (P_β’_–L_α_); 37.6–46.0/46.5–55.0 °C (ordered and disordered L_α_), respectively. 

Preliminary studies made recently by our research group are related to the individual characterization of the functional groups using Raman spectroscopy, these studies permit the quantification of the order/disorder of the DPPC multilayer. The samples analyzed in that study were deposited over hydrophilic and hydrophobic substrates—silicon wafer—using Langmuir-Blodgett technique. Thus, shifting and intensity changes in particular vibration bands of the Raman spectrum confirm molecular water presence between DPPC layers and certain interdigitation between aliphatic chains of the phospholipids [24]. 

According to the literature, DSPC has a sharp phase transition near to ~54–55 °C [52]. This system presents a particular thermal behavior, which can be observed in Figure 2b. At 21.3 °C, initial point, a thickness of ~6.4 nm was detected, value that slightly decreases to 6.1 nm when the sample reaches 33.0 °C, in this interval two type of domains can be clearly distinguished; the first corresponds to lipids that are fully stretched but with certain free movement in their tails (no overlapping between neighbor lipid tails; ordered domain) and disordered domains of their aliphatic chains (scheme Figure 2b) [53,54]. Over this temperature an abrupt decrease in thickness becomes detectable (Δ thickness: 1.8 nm), behavior that is intrinsically related—at this temperature range—to the fact that lipid tails enter into a semi-random organization, indicative of a typical lamellar liquid phase (L_α_ ordered); similar studies have been carried out by the research group of Qin et al. [54]. Finally, a thickness stabilization at 53.4 °C is observable, pointing that random liquid phase (L_α_ disordered) was reached. The lateral distance between the tails of the phospholipids that form the bilayer increase progressively when the films are heated under controlled cycles [14,16,17,24]. Studies performed using SPR are concordant with the results obtained by ellipsometry, showing a proportional and highly similar behavior than those plots but with abrupt and sharp steps that are indicative of phase transitions in the phospholipid bilayer. However, these phase transition temperatures are not indicated in this manuscript for all of the compounds analyzed, due to the high amount of information; instead only SPR measurements for some of the most relevant mixtures are showed. 

### 3.3. Phospholipid Binary Mixture—Heterogeneous Membrane

Two PC-based phospholipids (DPPC and DSPC), whose thermal behaviors are shown in Figure 2, were mixed with DMPC and DOPC (Figure 3 and Figure 4) at different molar ratios with the aim of generating heterogeneous membranes and thus to observe their stability respect to controlled temperature changes. These phospholipids were mixed with DPPC and DSPC due to their low thermal stability as bare compounds. As the first example, a DMPC bilayer undergoes to a pre-transition at 13.9 °C from the lamellar gel (L_β’_) to the ripple gel (P_β’_) phase, and subsequently, to the main transition at 23.9 °C from the P_β’_ to the liquid crystalline (L_α_) phase [55]. Additionally, the main phase transition of the DOPC bilayer is founded between −21.0 °C and −11.8 °C [56]. 

The molar ratios used were: (DPPC or DSPC):DMPC (2:1) and (DPPC or DSPC):DOPC (7:1), these proportions were proposed considering that longer hydrocarbon chains have higher T_m_ than those with shorter chains, therefore DPPC (T_m_ ~41.3 °C) and DSPC (T_m_ ~55 °C) were utilized for increasing the mixture’s thermal stability. Additionally, the low amount of DOPC (T_m_ ~−22.0 °C) in the reaction mixture is related to the fact that it possesses a double bond in both tails, being particularly susceptible to hydrolysis or oxidation, influencing the thermal behavior of the entire bilayer [57]. Finally, it is important to mention that the molar ratio of binary mixtures was chosen in order to produce broad and well define phase transitions. 

Figure 3a shows the results obtained from ellipsometric studies for mixture DPPC:DMPC (2:1). This binary compound is denominated “ideal” because their surfactants have similar chemical structures, so it presents a high interaction between their components [58]. This bilayer displays an initial thickness of 6.9 nm at 21.2 °C, this value decay to ~6.1 nm at 55.0 °C (total differential thickness: 0.8 nm). The bilayer thickness of this membrane increases compared with pure DPPC (Figure 2a). 

The phospholipid with the lowest main phase transition (DMPC) stays in the liquid phase at room temperature since the other phospholipid (DPPC) stays in their solid (ordered) state. Phase separation of these compounds is mainly related to their difference in aliphatic chain lengths. It should be noted that the phospholipids are heterogeneously distributed into the artificial membrane, generating two well-separated domains between (Figure 3a) [59]. 

The phases and phase transitions of this particular mixture can be located at: 21.2–23.0 °C (P_β’_ of DMPC + L_C_ of DPPC); 23.9–26.7 °C (ordered L_α_ of DMPC + L_C_ of DPPC, Figure 2a); 27.3–28.2 °C (disordered L_α_ of DMPC + L_C_–L_β’_ of DPPC); 28.7–31.5 °C (L_β’_ of DPPC); 32.2–33.6 °C (L_β’_–P_β’_ of DPPC); 34.3–38.5 °C (1st P_β’_ of DPPC); 39.1–41.2 °C (2nd P_β’_ of DPPC); 41.9–46.0 °C (P_β’_–L_α_ of DPPC); 46.7–48.1 °C (ordered L_α_ of DPPC) and 49.4–55.0 °C (disordered L_α_ of DPPC). Two different ripple-gel phases could be attributed according to the small variations encountered in plot curvature (thickness changes)—the aliphatic chains can be both tilted or not tilted [60]. 

Additionally, the results obtained by SPR are: 20.9–23.3 °C (P_β’_ of DMPC + L_C_ of DPPC); 24.5–26.7 °C (ordered L_α_ of DMPC + L_C_ of DPPC); 27.3–28.7 (disordered L_α_ of DMPC + L_C_–L_β’_ of DPPC); 29.3–33.6 °C (L_β’_ of DPPC); 34.2–37.6 °C (L_β’_–P_β’_ of DPPC); 38.0–42.1 °C (P_β’_ of DPPC); 42.4–43.9 °C (P_β’_–L_α_ of DPPC); 45.3–49.5 °C (ordered L_α_ of DPPC) and 50.2–55.0 °C (disordered L_α_ of DPPC). These data only show one ripple-phase, different to the case analyzed before. This fact can be explained by the more constrained sample setup compared to that used in the ellipsometry assembly, in this case, the prism on the top of the sample produces a certain mobility impediment for the bilayer. 

The difference between chain lengths for DPPC (16 C aliphatic chains) and DMPC (14 C aliphatic chains) affect the reorganization of the molecules within the bilayer, process which is associated with an increase in their thickness. Previous studies made by our research group have been related to X- or Y-type DPPC bilayers deposited over a silicon wafer; concluding that the orientation of the phospholipids affects the molecular inclination degree, mobility, and stability of phases and their respective phase transitions [24]. 

Figure 3b illustrates the effect of a cis-double bond present in the acyl chains of one phospholipidic component into the mixture. The initial bilayer thickness is 5.7 nm at 20.1 °C, that then decay to 5.2 nm at 55.0 °C (differential thickness: 0.5 nm). Thus, the detected phases or phase transitions of the mixture composed by DPPC:DOPC (7:1) are: 20.1–21.8 °C (disordered L_α_ of DOPC + L_C_ of DPPC); 22.3–25.3 °C (L_C_ of DPPC); 25.9–27.3 °C (L_C_–L_β’_ of DPPC); 27.9–32.3 °C (L_β’_ of DPPC, Figure 2a); 32.9–34.9 °C (L_β’_–P_β’_ of DPPC); 35.6–40.5 °C (P_β’_ of DPPC); 41.3–43.2 °C (P_β’_–L_α_ of DPPC); 43.9–48.8 °C (ordered L_α_ of DPPC); 49.5–55.0 °C (disordered L_α_ of DPPC). Similar results were obtained through SPR technique: 22.7–31.4 °C (disordered L_α_ of DOPC + L_C_ and L_β’_ of DPPC); 32.2–37.6 °C (L_β’_–P_β’_ of DPPC); 38.2–42.8 °C (P_β’_ of DPPC); 43.6–45.0 °C (P_β’_–L_α_ of DPPC); 45.5–50.3 °C (ordered L_α_ of DPPC); 51.1–55.0 °C (disordered L_α_ of DPPC).

The gel to liquid phase transition has high solidarity, in other words, the molecules cooperate each other with the aim of generating new motional degrees of freedom; when one molecule pick up motional energy, then other nearby molecules enter into this state easier, this is the reason for the occurrence of the ripple phase [61]. 

The DSPC and DMPC form a nonideal mixture, that is, there is a broad gel-fluid coexistence region [62]. Figure 4a showed this characteristic for the bilayer, below 54.3 °C a non-random lateral distribution of both components are displayed, therefore it is possible to individually study the phases and phase transitions of each phospholipid. In this case, the total differential thickness is ~0.2 nm (23.8–55.0 °C). Between 23.8 °C and 35.6 °C is possible to observe a ripple to liquid (disorder) transition and gel transition of both components (L_β’_ of DSPC + L_α_ of DMPC, see scheme in the Figure 4a). When the temperature rises, different phases, and transitions of these compound mixtures, are observed according to the following temperature limits: 36.3–39.9 (L_β’_–P_β’_ of DSPC); 40.4–44.8 °C (P_β’_ of DSPC); 45.3–47.4 °C (P_β’_–L_α_ of DSPC); 48.1–55.0 °C (L_α_ of DSPC). 

Figure 4b shows a total thickness difference of 1.3 nm (between 21.6 °C and 54.2 °C). From 21.6 °C to 50.8 °C the system formed by the mixture DSPC:DOPC (7:1) presents minimal changes in their thickness, making it difficult to locate the limits of each phase. Over this temperature, it is possible to find a slight thickness stability that possesses a high associated error (standard deviation), this effect could be probably attributed to the transition P_β’_–L_α_ and to the L_α_ phase of the DSPC. Similar results were obtained through the SPR technique, demonstrating a clear mixture of phases/phase transitions that are concordant with the results shown by ellipsometry. 

### 3.4. AFM and Force Spectroscopy Measures

**Figure 5:**
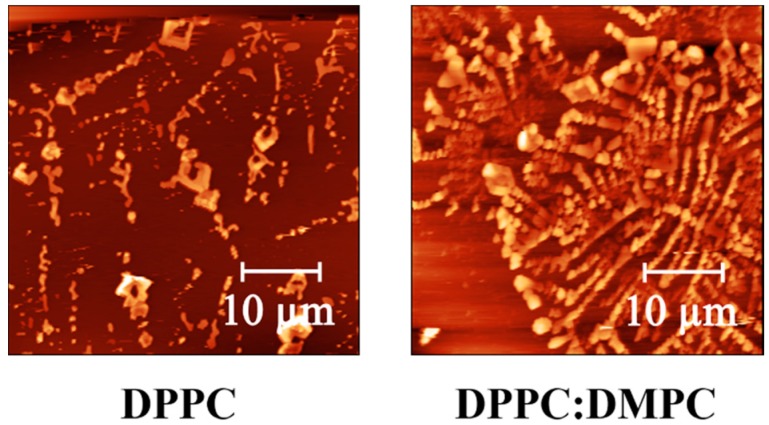
Atomic Force Microscope (AFM) micrographs of the samples DPPC and DPPC:DMPC (2:1), respectively.

In general, the bilayer thermal behavior can also be explained via the mechanical properties, such as elastic modulus (E), of the systems formed through Langmuir-Blodgett deposition. In order to test this hypothesis, force spectroscopy curves were measured using an AFM system several times (10 times) at different points (50 points) in sample triplicates (in total 500 measures per sample). Tip indentation process records the distance between the probe and the sample surface against the deflection of the cantilever, which permits to estimate the force exerted by the tip using their spring constant. Through the analysis of this data it is possible to determine the elastic Young’s modulus (E) of the surface using the Hertz model for the spherical tip over a smooth soft surface. The protocol is extensively explained in the study performed by the group of Roa et al. [41]. In Figure 5 it is possible to observe some of the AFM micrographs taken. The samples show in general a smooth surface with some clusters isolated, characteristic of homogeneous bilayer depositions. The micrographs shown next correspond to the borders of the samples in where is possible to observe the characteristic dragonflies formed by alkenes chains (phospholipid chains) using Langmuir-Blodgett technique [63]. 

Force spectroscopy was performed on the most relevant samples (DPPC, DPPC:DMPC (2:1) and DPPC:DOPC (7:1)) in order to understand their mechanical behavior. The results obtained are in concordance to similar studies performed by different research groups [64,65]. DPPC bilayer has a Young modulus of 51.32 ± 0.13 MPa, DPPC:DMPC present a modulus of 73.62 ± 0.16 MPa and DPPC:DOPC a stiffness of 48.26 ± 0.22 MPa. These results show that the mixture of DPPC:DMPC present higher Young modulus than the bilayer formed by bare DPPC, indicating that the insertion of DMPC into the mixture produces a stiffer bilayer. This mechanical impediment can explain the slight thickness changes that present in the sample DPPC:DMPC during thermal cycles in the ellipsometric measurements, compared to the abrupt variation suffered by DPPC sample, changes that are characteristic of phase transitions. 

Finally, the insertion of DOPC into the mixture of DPPC does not greatly affect the stiffness of the bilayer due to the low amount of DOPC included, this behavior, it seems, is also reflected in the similar phase transition temperatures found in this study for these two types of samples. 

In Table 2 it is possible to observe the variation in bilayer thickness for each compound analyzed in this study, the difference of bilayer thicknesses was calculated considering the initial (20–21 °C) and final (55 °C) values.

DPPC (16 C aliphatic chain) and DMPC (14 C aliphatic chain) mixture show an initial thickness of 6.9 nm, observing a considerable increase in the initial DPPC bilayer thickness (5.6 nm); this behavior is probably related to the reorganization of the molecules into the bilayer, in other words, the bilayer disruption creates extra free space into the planar membrane that allows the interaction with molecular water or phospholipid molecules overlapping. On the other hand, the small amount of DOPC into the mixture does not affect considerably the bilayer initial thickness (5.7 nm, Figure 3b). The chemical structural difference between DSPC and DOPC affects mainly the thermal bilayer stability (Figure 4b), producing difficulties at the moment of detecting the phase occurrence. 

The sample composed of pure DSPC (18 C aliphatic chain) shows an initial bilayer thickness of 6.4 nm with larger differences compared to the DPPC, behavior that could be attributed to their chain length and to the bilayer structuration. Additionally, the insertion of DMPC molecules into the mixture affects the mobility and the organization of the bilayer, showing a decrease in their thickness; opposite behavior to that observed for the case of DPPC:DOPC (7:1).

It is possible to analyze the effect that produces the insertion of different phospholipids into the system using the experimental design theory based on statistical methods like Yates algorithm for 2k factorial designs. Performing this procedure, the most relevant effect corresponds to the insertion of DMPC into the bilayer forming by either DPPC or DSPC. The most important variation in the characteristics analyzed (initial thickness and the total thickness difference) are produced with DMPC insertion. In the first case (DPPC), an increase of both characteristics was detected, and in the other case (DSPC) a decrease is produced due to the insertion of DMPC. This response can be explained due to binary lipid mixture generation, that possesses differences in their aliphatic chain lengths, showing the coexistence of two thermal behaviors due to non-ideal mixing; this effect is denominated dynamic lateral phase separation [66].

Finally, the small amount of DOPC into the DPPC or DSPC mixture, do not affect the initial or total difference thickness, but clearly, induce phase transitions in the systems analyzed. It is noteworthy that DOPC has two 18C with one unsaturation (18:1) and DPPC has two 16C fully saturated hydrocarbon chains. These structural differences account not only for the different transition temperatures, more densely packed and gel-like ordered structure for DPPC, and liquid-like behavior for DOPC at room temperature but also for other physicochemical properties, including differences in breakthrough forces, response to detergent addition, and so on. 

## 4. Conclusions

Thermal stability of the phospholipid bilayer composed of pure phosphatidylcholine-based molecules and certain mixtures with similar structure were analyzed through slight changes in optical and physical parameters obtained by ellipsometry and SPR techniques (real-time dynamic response). These changes are associated with negligible molecular movement into the layers, interdigitation, and so on. Thus, thickness variation and amplitude response under heating cycles were associated with phases and phase transition temperatures for each surfactant. All these studies were carried out using planar artificial films, that is, bilayer deposited over a silicon substrate through Langmuir-Blodgett method. This procedure creates artificial biomembranes that mimic or emulate the structure (and possibly, the behavior) of the fluid mosaic model of the cell membrane. 

Phospholipids with longer aliphatic chains (DSPC, 18C aliphatic chain) show higher transition temperatures. However, the insertion of an unsaturated molecule (double bond) into the structure (DOPC) produce a declination in the thermal stability of the bilayer. Additionally, binary lipid mixture membranes—with differences in aliphatic chain length—show the coexistence of two thermal regions into the bilayer structure due to non-ideal mixing between these two kinds of molecules. The insertion of DMPC (14C aliphatic chain) into the bilayer does not present important repercussions in the thermal behavior of DPPC or DSPC, but strongly affects the arrangement of the phospholipids in the bilayer due to the changes in their mechanical response (Young modulus) producing a spatial impediment for phase transition occurrence. The thermal behavior of the artificial membranes was also corroborated with SPR, the results are similar to that observed in the ellipsometric data.

As a conclusion, these studies indicated that the presence of heterogeneous (non-ideal) membrane structures that possess co-existing domains in the crystalline/gel-fluid phase are intimately related to their chemical and mechanical properties, such as chain length and Young modulus. 

## Figures and Tables

**Table 1:** Refractive indexes measured by Abbe refractometer in solution and in solid (film) used for ellipsometry model.

Material	Refractive Index (Solution)	Refractive Index (Film)	Extinction Coefficient
Silicon (Si100)	N/A	3.870	0.019
Silicon Dioxide (SiO2)	N/A	1.462 ± 0.025	N/A
DPPC	1.489 ± 0.063	1.484 ± 0.058	N/A
DSPC	1.426 ± 0.089	1.439 ± 0.086	N/A
DPPC:DMPC (2:1)	1.461 ± 0.068	1.488 ± 0.072	N/A
DPPC:DOPC (7:1)	1.459 ± 0.078	1.481 ± 0.067	N/A

**Table 2:** Total thickness bilayer variation for each phospholipid mixture.

	Thickness (nm)		
	21 °C	55.0 °C	Difference (Δ)
DPPC	5.6 ± 0.2	5.0 ± 0.3	~0.6
DSPC	6.4 ± 0.3	4.6 ± 0.4	~1.8
DPPC:DMPC (2:1)	6.9 ± 0.2	6.1 ± 0.5	~0.8
DPPC:DOPC (7:1)	5.7 ± 0.1	5.2 ± 0.6	~0.5
DSPC:DMPC (2:1)	4.2 ± 0.3 *	4.0 ± 0.4	~0.2
DSPC:DOPC (7:1)	5.1 ± 0.2	3.8 ± 0.5	~1.3

* Value took at 23.8 °C.
